# Education Technology in Orthodontics and Paediatric Dentistry during the COVID-19 Pandemic: A Systematic Review

**DOI:** 10.3390/ijerph18116056

**Published:** 2021-06-04

**Authors:** Assunta Patano, Nunzio Cirulli, Matteo Beretta, Paola Plantamura, Alessio Danilo Inchingolo, Angelo Michele Inchingolo, Ioana Roxana Bordea, Giuseppina Malcangi, Grazia Marinelli, Antonio Scarano, Felice Lorusso, Francesco Inchingolo, Gianna Dipalma

**Affiliations:** 1 Department of Interdisciplinary Medicine, University of Bari “Aldo Moro”, 70124 Bari, Italy; assuntapatano@gmail.com (A.P.); dottore@studiocirulli.it (N.C.); ad.inchingolo@libero.it (A.D.I.); angeloinchingolo@gmail.com (A.M.I.); graziamarinelli@live.it (G.M.); francesco.inchingolo@uniba.it (F.I.); giannadipalma@tiscali.it (G.D.); 2 Private Practice in Bari, 70121 Bari, Italy; 3 Digital Dentistry, Private in Varese, 21100 Varese, Italy; teoberet@libero.it; 4 Department of Computer Science, University of Bari “Aldo Moro”, 70121 Bari, Italy; paola.plantamura@uniba.it; 5 Department of Oral Rehabilitation, Faculty of Dentistry, Iuliu Hațieganu University of Medicine and Pharmacy, 400012 Cluj-Napoca, Romania; 6 Department of Innovative Technologies in Medicine and Dentistry, University of Chieti-Pescara, 66100 Chieti, Italy; ascarano@unich.it

**Keywords:** orthodontics, paediatric dentistry, e-learning, distance learning, virtual learning, pandemic, COVID-19, SARS-CoV-2

## Abstract

Over the last decade, medical education changed from traditional teaching methods to telematic and networking scholar and e-learning approach. The objective of the present systematic review was to evaluate the effectiveness and teachers/student’s acceptability of e-learning applied to the field of orthodontics and paediatric dentistry. A database search of the literature was conducted on PubMed and Embase databases from January 2005 to May 2021. A total of 172 articles were identified by the electronic search, while a total of 32 papers were selected for qualitative analysis. Overall, 19 articles investigated the effectiveness of e-learning, and no difference of acceptability was reported between e-learning and traditional methods for a wide part of the articles selected. A total of 25 papers provided a satisfaction questionnaire for learners and all were positive in their attitude towards e-learning. The results showed that e-learning is an effective method of instruction, complementing the traditional teaching methods, and learners had a positive attitude and perception. The evidence of the present study reported a high level of acceptability and knowledge level of e-learning techniques, compared to frontal lecture methods, in the fields of orthodontics and paediatric dentistry.

## 1. Introduction

**Figure 1:**
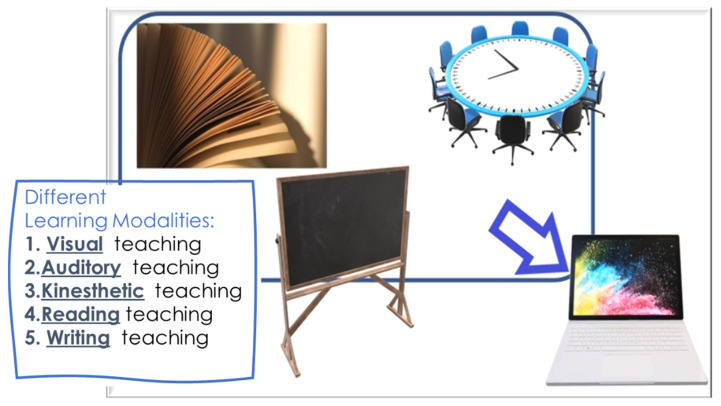
Transition from traditional learning to e-learning.

One of the most important developments in recent years is the evolution of technology, which has changed many aspects of our everyday life: our means of communication, information retrieval, even the way we spend our free time (e.g., computer games) [1,2] The importance of technology became even more evident during the COVID-19 pandemic that has had a massive impact on people’s lives and habits. Restrictions limited people’s mobility while remote working, e-learning, and online platforms started to grow, along with online leisure solutions, such as gaming and video streaming [3,4,5,6,7,8,9]. The COVID-19 pandemic debuted in December 2019, and since then, changed the lives of every person around the world [10,11,12,13,14,15,16,17,18,19,20,21,22,23]. During this pandemic, physical distancing measures were imposed, and consequently, the education field had to adapt and transition to online platforms because this type of learning allows participation from all over the world to a meeting, webinar, course, or class [24,25,26,27,28,29,30,31]. The educational sector globally has shifted from traditional classroom teaching towards e-learning since most countries around the world experienced the temporary closure of all educational institutions in order to contain the spread of the pandemic [32]. (Figure 1).

**Figure 2:**
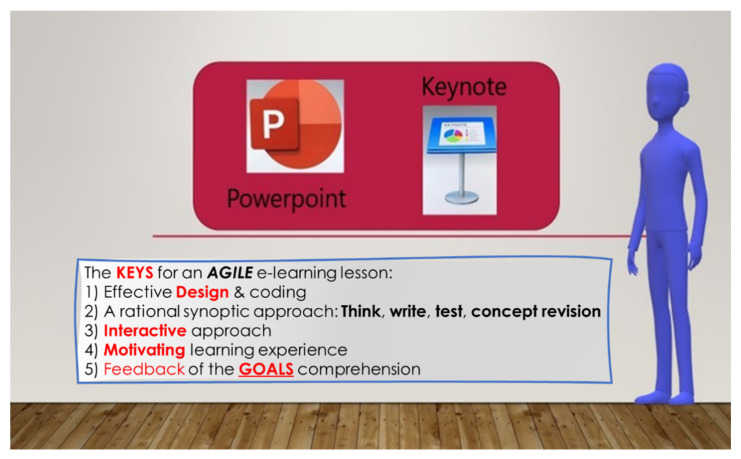
Example of two of the most employed software to create educational content.

Over the last decades, e-learning has rapidly expanded in medical education, health promotion, patients, and medical education that take advantage of a useful networking flow and flexibility of the communication system [33,34,35]. Moreover, healthcare education faces higher challenges determined by an increase in students’ access to post-degree courses and specialisation that requires novel strategies to improve the quality of the scholarship and the didactic level [35,36,37]. E-learning allows students to learn anywhere and anytime outside the classroom, overcomes the shortages of teachers, and promotes learner’s motivation, cognitive effectiveness, and flexibility, leading to a shift from passive, teacher-centred learning to active, student-centred learning. It is affordable, saves time, and reduces costs [27,38]. As stated by Zhang et al., the mass quarantine caused a feeling of fear [39], and Hasan et al. showed in their study that there can be a strong relationship between the e-learning-related breakdowns and the psychological status of the student [40]. In the past, education occurred by means of textbooks, handouts, and notes taken during courses. E-learning enables teachers to represent the information using media in the form of text, images, animation, video, and audio [41,42] (Figure 2).

**Figure 3:**
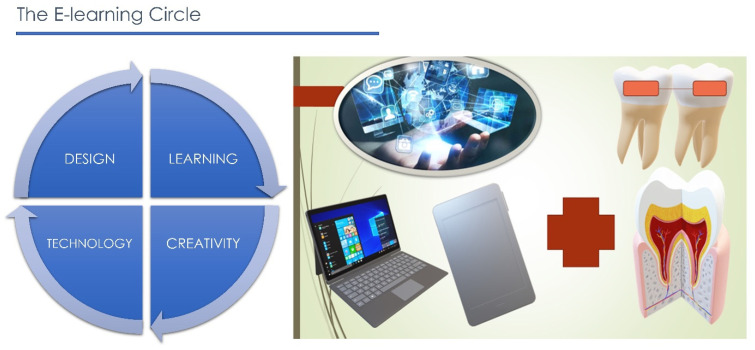
E-learning combination between telecommunication and medical field.

There are several important factors that need to be considered for the success of e-learning: human factors pertaining to the instructors, the instructors’ and students’ technical competency, the instructors’ and students’ attitudes, the level of collaboration, and the technical support [43]. E-learning is a generic term that refers to electronically supported learning and teaching. It includes a variety of modalities and terms such as web-based learning, online learning, computer-assisted instruction, internet-based learning, distance learning, and virtual learning [27,44,45]. E-learning can be synchronous or asynchronous. Synchronous e-learning requires participants to log on at the same time and allows students to interact with each other and their teachers during the lessons. Asynchronous e-learning refers to e-learning that is ‘pre-recorded’ or available to students at any time of the day, potentially from any place [46]. Numerous studies conducted on e-learning in medical education showed that participants considered e-learning as an effective reinforcing method for medical training, without missing the traditional style of teaching [47,48,49,50]. A combination of traditional face-to-face learning and e-learning is called blended learning. The main advantage of blended learning is that it integrates the strengths of synchronous traditional face-to-face teaching and asynchronous/synchronous web-based learning activities [1,2]. Blended learning increases the learning flexibility in a demand-driven educational environment while maintaining the personal contact of the traditional face-to-face teaching, enhancing the classroom experience, and improving effectiveness and efficiencies by reducing lecture time [51,52]. It has been suggested that blended learning, i.e., e-learning and virtual learning environments mixed with a traditional lecture style, improve competencies and core knowledge of students [53]. During the last decade, the large use of smartphones and the internet has fostered widespread use of social media. Social networks such as Facebook, Twitter, YouTube, Google Drive allow people from different backgrounds to communicate and collaborate with other users across the world [53,54,55]. The growing interest in social media among students and the ubiquitous distribution of portable electronic devices has led instructors to improve their teaching and learning through combining social media applications, online platforms, and mobile technologies (Figure 3) [56,57,58,59].

**Figure 4:**
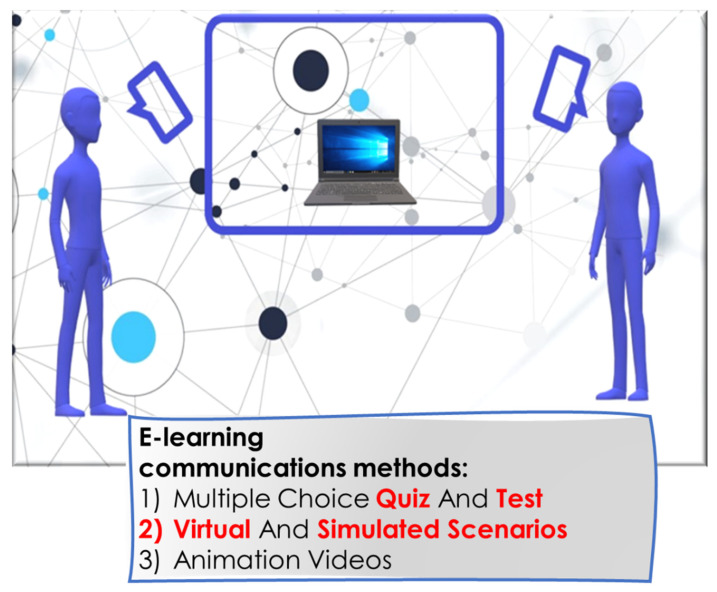
Webinars on multiple topics held online.

Mobile learning occurs when the student is not in a permanent and fixed location or if he is using mobile learning technologies. It is considered a part of e-learning [60]. Mobile devices such as smartphones, tablets, and laptop computers enable users to learn at any place and time, in different contexts and situations, and by interacting with others [30,61,62,63,64,65,66,67]. Furthermore, the growing availability of smartphones and tablets allows the mobile use of augmented reality in medical education [68]. The development of technology has affected even the field of dentistry, and numerous studies have been conducted on digital development in dental education [69,70,71,72,73,74,75,76,77]. In recent years, the diffusion of social media activities and web-based technologies has potentiated the information flow shared in several medical contexts and also in dental field education [78]. This form of interaction is useful at many different levels, such as for the education of undergraduate students, to enhance the expertise of younger dentists, in addition to improving the learning processes of experienced clinicians (Figure 4) [78,79,80,81,82,83,84,85,86].

## 2. Materials and Methods

This systematic review was conducted according to the Preferred Reporting Items for Systematic Reviews and Meta-Analyses (PRISMA) statement [87]. The article screening, selection for eligibility, and qualitative analysis of the study data were conducted by two independent paired reviewers (A.P., F.I.). If any disagreement occurred and unresolved issues were solved by consulting a third reviewer (F.L.). The screening phase was conducted on electronic databases which evaluated the manuscript title and abstract. The full text was collected for all identified articles in order to evaluate the qualitative analysis eligibility.

### 2.1. Eligibility Criteria

Articles in which the objective was to determine the effectiveness and acceptability of e-learning or to compare e-learning with conventional teaching methods were considered.

The inclusion criteria were based on the PICOT question guidelines [88,89]:

- Population: students from graduate and postgraduate courses in orthodontics or paediatric dentistry; university staff; dentists who used e-learning tools to update their knowledge and continuing formation;
- Intervention: use of virtual environments for learning;
- Comparison: traditional classroom learning; traditional methods of instruction through the lectures, the clinical or laboratory demonstration, tutorial, text-or note-based learning;
- Outcome: effectiveness and acceptability of e-learning;
- Types of study to be included: cohort, observational, retrospective, or prospective study with emerging effectiveness in the last 16 years.

The inclusion filters were cohort, observational, retrospective, or prospective studies regarding the e-learning and virtual learning performance of dentistry specialisation of student scholars.

### 2.2. Exclusion Criteria

Reviews, letters, conference readings, editorial, personal opinion, and studies without abstracts were excluded. We limited the searches to articles that were published in the last 16 years.

### 2.3. Information Sources

A systematic electronic search on PubMed and EMBASE databases was performed limited to English language articles published between January 2005 and May 2021. A preliminary search was conducted by the Pubmed MeSH terms function of medical subject headings to identify the most appropriate descriptors and qualifiers of the present research topic to use for the Boolean search. The EMBASE Boolean search has been conducted by Emtree search algorithm. We used the following keywords: orthodontics, pedodontics, paediatric dentistry, e-learning, distance learning, web-based learning, and virtual learning. The search algorithm was (orthodontics OR pedodontics OR paediatric dentistry) AND (e-learning OR distance learning [Mesh] OR web-based learning [Mesh] OR virtual learning [Mesh:NoExp]). The final search was run on 30 April 2021.

### 2.4. Risk of Bias Assessment

The assessment of the risk of bias of the included studies was independent and in duplicate in accordance with the EPOC guidelines [90]. A contribution was considered at high risk of bias in case of high/unclear risk of the ‘random sequence generation’ criterion. The risk of bias assessment was performed by a special data form by the software package Review Manager RevMan V 5.1 (The Nordic Cochrane Centre, The Cochrane Collaboration, Copenhagen, Danmark). 

### 2.5. Study Selection

Study selection was accomplished through three different levels as follows:

(1) Screening: all articles retrieved from these initial search criteria were subjected to a screening process by reading titles and abstracts;
(2) Eligibility: in a second phase, the eligibility criteria were applied to the full-text version of the selected articles;
(3) Inclusion: the remaining articles were included in the qualitative synthesis.

The following data were extracted from each study: year of publication, country and setting of the study, aims of the research, number of participants, e-learning teaching method, comparison with traditional teaching methods, effectiveness and acceptability to students, teachers, or private practitioners of e-learning.

## 3. Results

**Figure 5:**
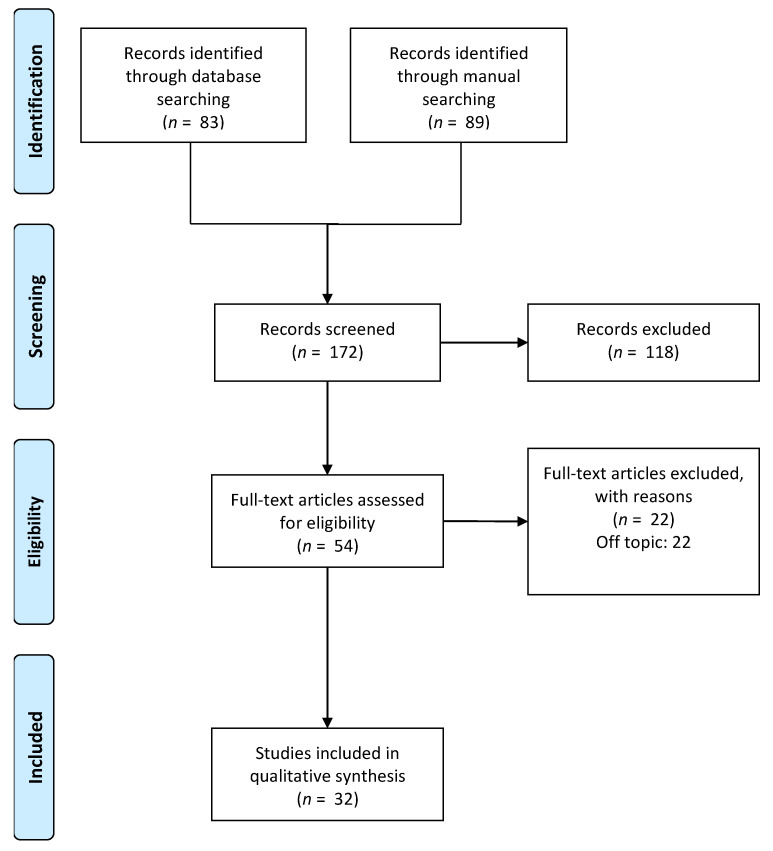
Selection of studies for the systematic review.

The article search identified 172 studies consisting of 83 papers from electronic database search and 89 contributions detected manually. After the initial screening identification process, a total of 54 articles met the inclusion criteria applied to the title and abstract assessment. The full texts were evaluated for the eligibility criteria, and a total of 32 papers were deemed suitable for inclusion in this review. The study selection process is illustrated in Figure 5. The included studies are summarised in Table 1.

### 3.1. Characteristics of the Studies Included

The studies were conducted in the United States (10), United Kingdom (8), Brazil (5), Germany (3), Australia (3), Greece (1), Iran (1), and Saudi Arabia (1). Among the selected studies, 20 analysed the use of the e-learning teaching method in orthodontic education, while 11 studies evaluated the efficacy of e-learning in paediatric dentistry. One study evaluated the effectiveness of web-based self-instruction in both orthodontics and paediatric dentistry. The sample size for the included studies ranged from 9 to 430 participants. Participants were mostly undergraduate and postgraduate students or faculty members. Three studies involved dentists working in PHC, and one study involved private orthodontists. Studies evaluated many different educational interventions of varying duration, frequency, and format. The delivery modes used to deliver the educational materials included CD-ROM, learning management systems (e.g., WebCT, Moodle, and Blackboard), DVD, web browsers, and virtual learning environments. The methods used for interaction between trainers were videoconference, telephone, internet chat, or e-mail. Additionally, 16 articles included a comparison between e-learning or blended learning and traditional teaching methods.

### 3.2. Risk of Bias Assessment

**Figure 6:**
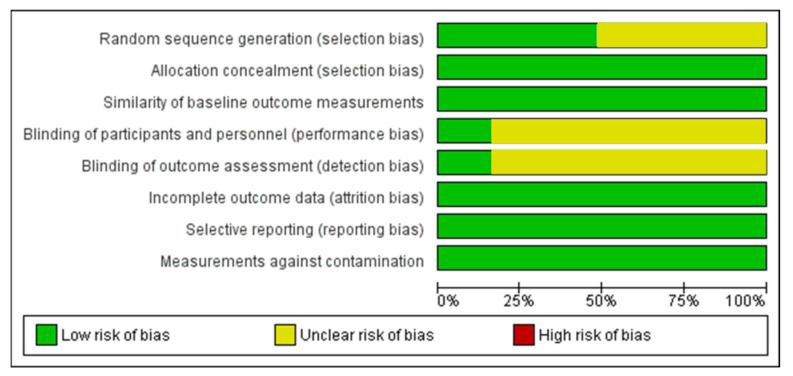
Graph of the risk of bias assessment of the included studies.

**Figure 7:**
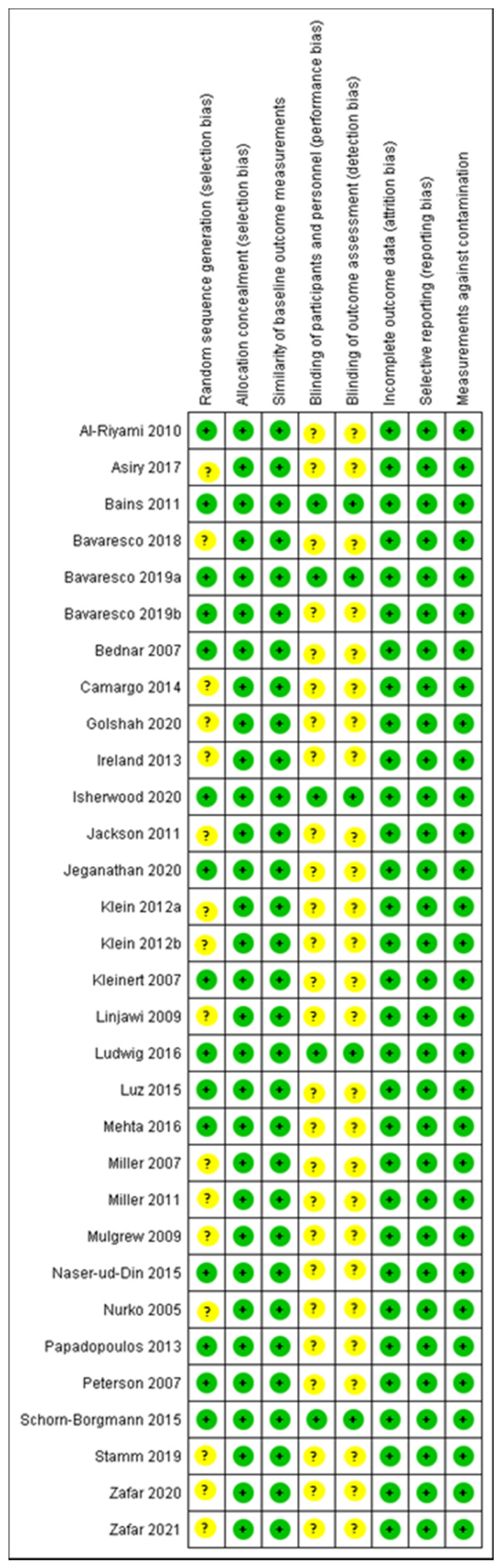
General summary of the risk of bias assessment of the included studies.

The outcome of the risk of bias assessment of the included articles was reported in Figure 6 and Figure 7. The 48.38% of articled reported a low bias of randomisation protocols, while the similarity of outcome measurements and selective reporting of outcomes presented a low risk of bias. The blinding approaches presented an unclear risk of bias of the studies selected. In most of the articles, the contamination bias was low.

### 3.3. Effectiveness and Acceptability of the E-Learning Methods

Two outcome measurements were considered in this review: effectiveness and acceptability. The effectiveness of e-learning was investigated in 19 studies evaluating the quantity of knowledge gain using multiple-choice questionnaires, open-ended questions, or practical exams. A significant improvement in participants’ knowledge after web-based courses was reported in eight studies. Mulgrew et al. concluded that travel commitments for trainees have reduced as a result of introducing the web-based resource but not as expected [97]. Camargo et al. found that graduate students finished the course with better performance than undergraduate students [107]. Of the 32 studies, 16 compared e-learning with traditional learning. In the majority of the studies, no difference was observed in knowledge gained between the two methods, whereas two studies concluded that e-learning was more effective than traditional methods. Papadopoulos et al. found a statistically significant difference between the group that used a virtual patient and the control group showing a gain in knowledge in the simulation group [106]. Luz et al. assessed that the ICDAS e-learning programme was more effective than traditional learning in improving dental students’ ability to use ICDAS [108]. Bains et al. compared e-learning with blended learning and face-to-face learning and he found that e-learning was less effective, while blended learning was the most preferred [99]. The changes in performance following learning were evaluated in five studies. Schorn-Borgmann et al. evaluated the performance of students in the construction of orthodontic appliances, and no significant improvement in the practical result was identified [110]. Ludwig at. al also failed to identify significant differences between face-to-face learning and the use of cephalometric imaging software [111], while Al-Riyami et al. found no difference in student performance in diagnosing TMD after VLE learning or face-to-face learning [98]. Luz et al. evaluated students’ performance in detecting dental caries [108]. Students’ acceptability was considered as an outcome in 25 studies. Seven of these studies mentioned that student satisfaction was evaluated with a Likert-scale questionnaire. The other studies used different types of questionnaires or surveys without mentioning the use of the Likert scale. All these studies reported a positive response from students when using online learning. In six studies, the students viewed online learning helpful as a supplement to their learning rather than a replacement for traditional teaching methods. Linjawi et al. stated that students responded ‘very positive’ to ‘positive’ for orthodontic e-course design, course delivery, and course outcome, but the orthodontic e-course was considered by most students as an adjunct and not a replacement of the traditional teaching methods [96]. Asiry found that few students preferred the online teaching method, and fewer students agreed to replace traditional lectures and live demonstrations with online tutorials, while most students preferred a combination of these teaching methods [113]. Mulgrew et al. concluded that despite the popularity of web-based learning resources, trainees continue to value the opportunity to interact face-to-face with their teachers [97]. Zafar et al. found in their study that 80% of the participants disagreed that virtual reality should replace conventional simulation [119]. In another article, Zafar et al. assessed that the use of VR simulation can be an additional tool that enhances students learning experience, without replacing traditional training methods [122]. According to the majority of studies, online courses were easy to access, well constructed, and understandable. However, Klein et al. found that the logistics of scheduling distant seminars, and uneven quality of the audio and video recordings were the major concerns of participants. They also assessed that students’ perceptions of the quality of the learning material were influenced by the depth of their preparation [103]. In the article of Peterson et al., students preferred the online textbook to traditional textbooks, but they had technical problems associated with online use of computers running obsolete (internet) browser software [93]. Bednar et al. stated that acceptability of the distance seminars appeared to be influenced by the instructor’s personality and teaching style, the seminar subject, and the residents’ technological level [92]. Only two studies evaluated the opinions of faculty members that showed a positive attitude towards e-learning. Klein et al. concluded that faculty members were somewhat more enthusiastic about the experience than were residents, and they would like to use this approach to distance learning again [104]. Mulgrew et al. found that the trainers felt that teaching has been more interactive and enjoyable since the introduction of the web-based learning resource even if they stated that it has changed but not reduced teaching commitments [97].

## 4. Discussion

To the best of our knowledge, this is the first systematic review examining the use of e-learning in paediatric dentistry, while several reviews have been published in orthodontics [123]. This review showed that the use of e-learning has a positive impact on healthcare education. The rationale of the present investigation considered only the bodies of evidence on e-learning methods in the last 16 years in accordance with the first worldwide expansion of scholarship using social media platforms, while Facebook reported, on 1 October 2005, a total of 21 universities in the UK and others around the world use the platform. This evidence is commonly considered the beginning of the social media application in a scholarly environment.

The limits of the present investigation regarded the several differences of learning methodologies, the wide heterogeneity of the study population (undergraduate students/specialisation-related courses/teachers), and the feedback measurements modalities of the acceptability and effectiveness level. According to these bias factors, a statistical consideration/meta-analysis approach was not applicable for the present investigation.

On the contrary, the rationale of the present study design offered the widest possible level of scholars, from novice/undergraduate students to those with advanced levels of expertise, not dispersing the sensitivity of the study.

Most studies reported a significant gain in knowledge after e-learning, which confirms that e-learning is effective in increasing knowledge after training in both orthodontics and paediatric dentistry. Studies that compared e-learning to traditional methods concluded that e-learning was at least as effective as traditional learning.

These results agreed with those of Lima et al. [124]. In a review, they evaluated the impact of tele-education in the field of orthodontics and concluded that orthodontic distance learning is an effective but complementary element, with no significant differences from the traditional methods of learning [124]. Kumar found that e-learning classes are at least as good as and/or better than face-to-face classroom learning and the blended approach which combines both traditional face-to-face learning and e-learning is the best method of teaching and learning [125]. Our secondary aim was to assess the acceptability of e-learning from students and teachers. This topic was explored in the interviews and questionnaires. The majority of participants considered e-learning to be effective and easy to use. According to Bednar et al., there are two benefits from using distance learning. It can enhance the experience of residents by exposing them to a variety of different thoughts, ideas, and other residents and instructors, and it can alleviate problems associated with decreasing numbers of experienced full-time faculty [92]. Many studies underlined the importance of interaction with faculty members. According to Camargo et al., interaction with tutors should provide motivation, guidance, and support to students. Klein et al. found that 92% of the participating residents thought the post-seminar discussion was an important part of the learning experience [103]. Furthermore, Miller et al. stated that participants preferred post-seminar videoconference in comparison with audio-only or chatroom interaction [101]. The studies we reviewed suggest that students prefer that online modules are used as a support to learning, and they dislike the replacement of traditional lectures with online instruction. In fact, a blended approach, mixing person-to-person contact with e-learning methods, seems the most preferred. Possible explanations could be as follows: (1) compared with traditional learning, blended learning allows students to review electronic materials as often as necessary; (2) compared with e-learning, blended learning learners are less likely to experience feelings of isolation or reduced interest in the subject matter [126].

A particular type of e-learning is virtual learning. Only four articles included in this review examined the use of virtual reality. Kleinert et al. described the use of an interactive, multimedia virtual patient module developed on compact disc (CD-ROM) to increase students’ competence in caring for children with developmental disabilities [95]. Papadopoulos et al. demonstrated that a paediatric dentistry virtual patient built in a virtual world offers significant learning potential when used as a supplement to the traditional teaching techniques [106]. This result agreed with Zafar et al. who assessed that the Simodont simulated learning environment could be used as an adjunct in training dental students for preclinical paediatric dentistry restorative exercises [119]. Finally, Zafar et al. [122] presented the use of a VR simulator tool for local anaesthesia teaching in paediatric dentistry.

Other studies have demonstrated the consistent efficacy of virtual patient programmes in a variety of educational fields, including clinical training in healthcare professions [127,128,129,130,131,132,133,134,135].

Our study has several limitations. There are many confounding factors in learning that were not controlled for in the studies, such as the level of motivation of the studies, previous knowledge, and teaching style of the educators. The protocol of the present review excluded studies with no abstract. Interventions, topics, durations, and settings were different for every study. Traditional evaluation methods such as written texts, questionnaires were used for evaluation knowledge gain. It is unclear to what extent these methods can measure the effectiveness of e-learning, and how they may have influenced the outcome.

The number of studies published on the use of e-learning, in comparison with traditional learning methods, was relatively limited. Other limitations were found in the selected studies, especially due to the failure to define the content quality and type of specific e-learning intervention being analysed.

Moreover, studies did not report motivations that led to choosing a specific teaching method.

Furthermore, we observed that the impression of the educator was evaluated in few studies.

## 5. Conclusions

The Sars-CoV-2 pandemic worldwide emergency produced deep modifications of the institutional educational system with increased use of the smart-working approach, e-learning platforms, and limited use of traditional methods of academic learning. Within the limits of the study, the effectiveness of the present investigation demonstrated that e-learning is effective as traditional classroom methods, and the learners in these studies reported positive attitudes about e-learning with a high level of efficacy and acceptability by the operators and students. More detailed studies are necessary to understand the integration of e-learning into the learning methods in academic institutions and the implementation of interactivity in learning environments of dental students with special attention to the practicing clinical decision-making skills and operative procedures.

## Figures and Tables

**Table 1:** Summary of the studies included in the systematic review.

Author Year	Country	Aim	Participants	Methods	Feedback Measurement	Traditional Method	Effectiveness and Acceptability
Nurko and Profitt (2005) [91]	USA	Effectiveness of Web-based self-instruction/small-group seminars for an orthodontics predoctoral course	Not mentioned	10 self-learning teaching modules (Macromedia Directors) 4 seminars for discussion	Final questionnaire; Likert scale	Yes	Effectiveness: web-based seminars were effective as traditional methods Acceptability: web-based seminars and small-group discussions are well accepted by students
Bednar et al. (2007) [92]	USA	Effectiveness and acceptability of Web-based instructor	45 residents and 4 faculty	(A) seminars of basic concepts and clinical underlying principles (B) clinical conferences and treatment plans evaluation (C) clinical theme seminars and treatment plans	Effectiveness: pre- and post-test scores Acceptability: Likert scale; open questions	No	Effectiveness: A statistically significant increase in test scores for participants in all groups and interactive groups. Acceptability: high scores in all groups and interactive group
Peterson et al. (2007) [93]	USA	Evaluate the dental student perceptions regarding Web-based education in paediatric dentistry	55 third-year undergraduate dental students	Students were surveyed regarding their use of the Atlas of paediatric dentistry as the textbook resource during a paediatric dentistry course.	Acceptability: questions regarded the students’ attitude toward the Atlas and online education	Yes	Acceptability: students preferred the online textbook to traditional textbooks. The complaints resulted from the use of home computers with slower connection speeds and a programming problem that made it difficult to access the quiz part of the book
Miller et al. (2007) [94]	USA	Evaluate the effectiveness of 3 methods of pre-recorded seminars in orthodontics	First-year residents from three universities	Recorded seminars and follow-up interaction for residents and practicing orthodontists via video conferencing, telephone, and internet chat with Net Meeting software (Microsoft, Bellevue, Wash)	Acceptability: closed- and open-ended questions	No	Acceptability: the use of distance learning methods mediated by recorded seminars and monitoring interactions is an accepted method for teaching orthodontics. Residents agreed that the overall experience was an effective and efficient way to learn. Videoconferencing received the highest ratings
Kleinert et al. (2007) [95]	USA	Examine the effectiveness of a CD-ROM virtual patient learning module	51 students	An interactive, multimedia, virtual patient module was designed and developed on compact disc (CD-ROM) to increase students’ competence in caring for children’s disabilities.	Effectiveness: pre- and post-test of knowledge Acceptability: Usability Scale	No	Effectiveness: significant results were obtained in knowledge Acceptability: participants reported overall satisfaction with the module
Linjawi et al. (2009) [96]	UK	To develop an online undergraduate orthodontic e-course and assess its success as a learning resource from the student’s perspective	64 third-year undergraduate students	E-course composed of modules, photo gallery, clinical consultations, glossary, list of readings and resources	Acceptability: feedback questionnaire (Likert scale)	No	High acceptability by the students that responded ‘positive’ for course design, course delivery, and course outcome. Web-based material as supplemental for traditional teaching methods
Mulgrew et al. (2009) [97]	UK	Evaluate the effects of a web-based modular teaching programme, housed with a virtual learning environment on orthodontic training	9 trainees (postgraduates) and 14 trainers	Modular teaching programme, housed with a VLE	Effectiveness and acceptability: semi-structured interviews and focus group	No	Effectiveness: Positive effects on postgraduate orthodontic teaching and learning. Travel commitments for trainees have been reduced, but not as expected and demands on academic staff have not reduced but have changed Acceptability: Improvements in the flexibility and efficiency of learning. Trainees continue to value the opportunity to interact face-to-face with their teachers
Al-Riyami et al. (2010) [98]	UK	Compare the instructional efficacy of an internet-based temporomandibular joint (TMJ) tutorial with a traditional seminar	30 orthodontic graduate students	Group 1: Moodle VLE tutorial followed by the face-to-face seminar Group 2: Face-to-face seminar followed by Moodle VLE tutorial	Effectiveness: at the end of the course students were required to examine a patient and diagnose their TMJ condition Acceptability: anonymous questionnaire based on the learning experiences	Yes	Effectiveness: no differences were found between either teaching modes, and both are equally effective at delivering information to students Acceptability: students had positive perceptions of VLE learning, and the feedback to this mode of teaching was comparable with more traditional methods of teaching
Bains et al. (2011) [99]	UK	Compare e-learning, face-to-face (F2FL) learning, and blended learning (BL) with respect to their effectiveness and students’ attitudes toward them	157 fourth-year undergraduate students (90 completed the study)	F2FL: teacher-led tutorial EL: online tutorial developed by a Senior Orthodontic Register using WebCT^®^ version 3.8 BL1: EL first then F2FL BL2: F2FL first then EL	Effectiveness and acceptability were immediately assessed with an MCQ.	Yes	Effectiveness: no statistically significant difference between F2FL and BL. EL alone was less effective Acceptability: students are generally positive about all three methods but BL is the most and F2FL the least accepted, while EL is the least preferred
Jackson et al. (2011) [100]	USA	Evaluate the effectiveness of self-texts as a component of web-based self-instruction in predoctoral orthodontics and paediatric dentistry	157 postgraduate students	Online teaching modules, seminar for discussion, and self-texts.	Effectiveness: free-response, multiple-choice tests and seminar participation, and exercises related to the course material.	No	Effectiveness: The correlations between frequency of self-test access and course average were positive but not statistically significant. Increased use of web-based self-tests may be correlated with more effective learning in predoctoral dental education and that dental students’ usage of resources for learning changes significantly over the course of their education
Miller et al. (2011) [101]	USA	Evaluate the effectiveness and acceptability of various forms of post-seminar feedback after distant residents viewed recorded interactive seminars	Orthodontic residents	25 seminars organised into 4 sequences: Sequences 1 and 3 (UNC): growth and development of the face and biomechanics Sequences 2 and 4 (OSU): diagnosis, treatment plan, and treatment of sequelae Four different methods of post-seminar interaction: local follow-up discussion, videoconference, teleconference, and no discussion.	Effectiveness: pre- and post-tests Acceptability: satisfaction questionnaire at the end of the course	No	Effectiveness: post-test scores improved in each seminars sequence Acceptability: residents agreed that the videos helped them understand the material better than the readings alone and improved their educational experience. Residents preferred real-time interaction and local follow-up discussion regarding the videoconference and teleconference
Klein et al. (2011) [102]	USA	Test the acceptability and perceived effectiveness of using recorded interactive seminars and video conference (through WebEx or Elluminate software) follow-up discussions for in-office continuing education	23 orthodontists in private practice	Four groups of practitioners were asked to prepare for view and then discuss previously recorded interactive seminars; a fifth group (5 previous participants) had live discussions of 3 topics without viewing a pre-recorded seminar.	Acceptability: Likert scale and open-ended responses	No	Acceptability: Some participants reported difficulties using the videoconference system the private practitioners enjoyed their experience with this in-office (or home) version of continuing education and found it an effective way to learn
Klein et al. (2012) [103]	USA	Determine programmatic interest in using distance learning, resident and faculty interest, and the seminars’ perceived usefulness	253 residents and 42 teachers	25 interactive orthodontic seminars in 4 topic sequences and post seminars discussions with faculty	Acceptability: survey, Likert scale	No	Acceptability: the blended approach to distance learning was judged to be effective and enjoyable; faculty members were somewhat more enthusiastic about the experience than residents. Both were close to neutral about whether residents about interactive seminars as vs. traditional classroom. The post-seminar discussions were rated highly positive by both faculty and residents
Klein et al.(2012) [104]	USA	Discuss the problems that are likely to be encountered in the use of blended distance learning in postdoctoral health science education and possible solutions	253 residents and 42 teachers	Discussion of the problems and solutions of the previous study 25 interactive orthodontic seminars in 4 topic sequences and post seminars discussions with faculty		No	Acceptability: the biggest problem was lack of remote-based resident preparation and expectation of a lecture rather than a seminar. The logistics of scheduling distant seminars and uneven quality of the audio and video recordings were the major concerns of both residents and faculty members
Ireland et al. (2013) [105]	UK	Evaluate Wiki topic teaching in postgraduate orthodontists	9 postgraduate orthodontists	Students were divided into three groups and wrote and presented a Wiki on three interrelated topics using Blackboard platform.	Acceptability: feedback questionnaires	No	Acceptability: students felt writing the Wikis was useful for teamwork, provided a more learner-centred approach, and was a welcome variation on traditional teaching methods
Papadopoulos et al. (2013) [106]	Greece	Design and evaluate a virtual patient as a supplemental teaching tool for paediatric dentistry	130 undergraduate students	Simulation group: virtual patient Control group	Effectiveness: knowledge questionnaire Acceptability: evaluation questionnaire	Yes	Effectiveness: a statistically significant difference between the two groups was found showing a gain in knowledge in the simulation group Acceptability: the majority of participants evaluated the simulation very positively
Camargo et al. (2014) [107]	Brazil	Evaluate e-learning strategy in teaching ART to undergraduate and graduate students	76 participants (38 undergraduate students and 38 paediatric dentistry students in specialisation course)	DVD training course in ART. These e-learning courses combined many resources, such as the ‘Virtual Man project’, clinical videos, interviews with ART experts, clinical pictures, and radiographs	Effectiveness: tests performed before and after the course	No	Effectiveness: all students significantly improved their performances after the e-learning course. The comparison of the final evaluation grades between the two groups showed a statistically significant difference, indicating that graduate students finished the course with better performance than undergraduate students
Luz et al. (2015) [108]	Brazil	Evaluate the effect of a digital learning tool on undergraduate students’ performance in detecting dental caries using ICDAS	39 undergraduate students	Group 1: ICDAS e-learning programme Group 2: ICDAS e-learning programme plus digital learning tool (DLT) Group 3: traditional learning	Effectiveness: twelve paediatric patients were examined by the students before and after the training sessions	Yes	Effectiveness: sensitivity was statistically significantly higher for G1 and G2. G2 showed a significant increase in sensitivity at the D2 and D3 thresholds
Naser-ud-Din (2015) [109]	Australia	To investigate learning styles and the acceptance of e-modules as part of postgraduate training	9 postgraduate orthodontists	Nine interactive modules on Software SBLi^®^ for orthodontics postgraduate training Acceptability: post- SBLi open-ended questionnaire		Yes	Acceptability: high acceptance rate. E-modules demonstrated high compatibility with the learning styles of the participants
Schorn-Borgmann et al. (2015) [110]	Germany	Evaluate the effect of online demonstrations concerning the quality of orthodontic appliances manufactured by undergraduate dental students	55 undergraduate students	Group I: conventional lectures and live demonstrations Group II conventional lectures and access to an online blog Group III: access to all the materials of Group I and II, plus access to the online video material	Effectiveness: at the end of the course three orthodontic appliances made by the students were scored by tutors	Yes	Effectiveness: concerning the different appliances made, there was no significant difference in the outcome scores between groups
Ludwig et al. (2016) [111]	Germany	Assess whether e-learning improves learning efficiency and compare an opposite programme to commercially available software	30 fifth-year undergraduate students	Group 1 (control): traditional teaching method on 10 cephalometric radiographs; 6 weeks of training Group 2: PowerPoint created by the authors for the study; study of 10 cephalometric radiographs Group 3: commercial software for cephalometric study on 10 radiographs	Effectiveness: identification of 30 anatomical points on two radiographs in 5 min Acceptability: the students were interviewed	Yes	Effectiveness: the best improvement of scores was achieved by group 2 (8.6 points) compared to group 1 (four points) and group 3 (2.8 points) Acceptability: students preferred the PPT created by the authors to the commercial software, which the students found difficult to manage
Mehta et al. (2016) [112]	UK	Assess the impact of e-learning on student learning experience and orthodontic knowledge	63 fourth-year undergraduate students	Intervention group: Six Orthodontic modules of videos and multiple-choice questions with feedback. The E-learning resource was available to the test group through the student Virtual Learning Environment (VLE), namely QMPlus Control group: traditional teaching method	Effectiveness: quizzes before and after the course (6 weeks) Acceptability: satisfaction questionnaire at the end of the course	Yes	Effectiveness: no significant difference was observed between the test group and the control group Acceptability: user satisfaction with the resource was very high
Asiry(2017) [113]	Saudi Arabia	Identify the readiness of students for online learning and measure the quality of online tutorials	70 students (57 completed the study)	Online flash lectures, procedural video illustrating laboratory steps in addition to traditional face-to-face lectures, and laboratory demonstrations during the preclinical orthodontic course. Online tutorial links we received through Twitter. Twitter and Google Moderator were used.	Acceptability: satisfaction questionnaire at the end of the course	Yes	Acceptability: few students preferred online flash lectures (31.5%) and procedural videos (17.1%). Fewer students (11.1% agree and 3.7% strongly agree) agreed to replace traditional lectures and live demonstrations by online tutorials. Most students (38.9% agree and 31.5% strongly agree) preferred a combination of the methods
Bavarescoet al. (2018) [114]	Brazil	Develop a distance learning course in paediatric dentistry	430 dentists working in PHC (220 completed the study)	Distance learning course of paediatric dentistry composed of five modules that were made available to participants weekly. Every video class was also recorded and edited and made available to participants of the course through the Moodle Platform used as a virtual learning environment	Effectiveness: pre- and post-course quizzes. Participants were also invited to respond to a quiz about their personal and professional profiles. The software and online research tool Survey-Monkey^®^ was used to deliver the quizzes	No	Effectiveness: from pre- to post-course, there was a significant improvement in participant quiz performance. The variables age, time since graduation, and time working at PHC presented a statistically significant difference when correlated the grade with the pre-test average, while this difference was not observed when these variables were correlated with the post-test average
Stamm et al. (2019) [115]	Germany	Assess the impact of a one-to-one tablet PC programme by analysing students’ learning skills	108 students attended a clinical orthodontic course	One-to-one Tablet PC (TPC) programme. The NDE scores of students who participated in the TPC programme (n = 53) were compared with the scores of 64 students who attended the orthodontic scores before TPC deployment	Effectiveness: National Dental Examination (NDE) in orthodontics that evaluated theoretical knowledge and motor skills. Acceptability: survey	No	Effectiveness: the NDE scores of theoretical knowledge increased significantly after TPC deployment, whereas the scores for manual skills remained on the same level Acceptability: students expectations concerning the TPC benefit in the orthodontic curriculum improved significantly by using these devices
Bavaresco et al. (2019) [116]	Brazil	Evaluate the performance of dentists working in primary healthcare (PHC) in a paediatric dentistry distance learning course	430 Dentists working in PHC (201 completed the study)	Distance learning course of paediatric dentistry composed of five modules that were made available to participants weekly through the Moodle Platform used as a virtual learning environment	Effectiveness: Post-module questionnaire Participants were also invited to respond to a quiz about their personal and professional profiles.	No	Effectiveness: high rates of correct answers were observed after the course. It was observed that training in a public institution and a longer time since graduation positively influenced the grades earned on the restorative dentistry and dental trauma questionnaires, respectively
Bavaresco et al. (2019) [117]	Brazil	Assess the level of satisfaction of dentists working in primary healthcare with a distance learning course in paediatric dentistry	430 Dentists working in PHC	Distance learning course of paediatric dentistry composed of five modules that were made available to participants weekly through the Moodle Platform used as a virtual learning environment	Acceptability: satisfaction questionnaire (Likert scale); (40 answered the satisfaction questionnaire; 31 completed the satisfaction and personal/professional profile questionnaires)	No	The participants were satisfied with the course and attributed positive values to the variables evaluated. However, no statistically significant association was found between student satisfaction and the grades they earned on the pre- and post-course questionnaires
Isherwood et al. (2020) [118]	UK	Compare the ‘flipped classroom’ method and traditional lecture-based teaching for undergraduate students orthodontic emergencies	61 undergraduate students	Conventional group: lectures Flipped group: videos via VLE	Effectiveness: 20 questions Acceptability: semi-structured, open-ended focus group interviews	Yes	Effectiveness: there was no significant difference between the groups Acceptability: students were very positive about flipped classroom method of teaching and there was a general consensus that it should be incorporated into the undergraduate curriculum
Zafar et al. (2020) [119]	Australia	Compare students’ perception of the preclinical paediatric dentistry training gained in Simodont and conventional simulation environment	100 undergraduates	Lectures followed by practice sessions on the Simodont and conventional pre-clinical simulation. The Moog Simodont Dental Trainer provides a virtual reality-based dental simulation environment for training students.	Acceptability: Likert scale	Yes	Acceptability: participants felt Simodont training facilitated their understanding of paediatric dentistry tasks but the majority of the students disagreed that Simodont should replace conventional simulation.
Jeganathan and Fleming (2020) [120]	UK	Describe the use of blended learning as a method of undergraduate orthodontic teaching delivery and assess its effectiveness.	70 fifth-year undergraduate students	Intervention group: blending learning. The E-learning resource was developed using proprietary E-learning software. Clinical cases with diagnostic, in-treatment and final photographs and radiographs were included. Interactive features included questions on radiographs and flow diagrams. Control group: traditional seminar teaching	Effectiveness: tests both before (T0) and after(T1) the study period Acceptability: post-intervention student satisfaction survey	Yes	Effectiveness: no differences in short-term knowledge gain between two groups of students randomly allocated to teachings using either a blended or traditional seminar teaching was identified Acceptability: high levels of learner satisfaction common to both approaches
Golshah et al. (2020) [121]	Iran	Compare the efficacy of smartphone-based mobile learning versus lecture-based learning for instruction of cephalometric landmark identification	53 undergraduate students (4th year)	Intervention group: smartphone application instruction Control group: traditional lecture-based instruction Effectiveness: two weeks after the instruction, dental students were asked to identify four cephalometric landmarks		Yes	Effectiveness: no significant difference was noted between the two groups
Zafar et al. (2021) [122]	Australia	Investigate dental student’s perception of dental local anaesthesia (LA) virtual reality (VR) simulation on a paediatric patient and determine whether this can improve students learning experience	71 students	LA VR simulator software Acceptability: pre- and post-training survey containing open-ended and Likert-scale questions		Yes	Acceptability: most of participants agreed that LA VR simulator improved their knowledge of anatomical landmarks and added value compared with traditional LA teaching method

## Data Availability

All experimental data to support the findings of this study are available contacting the corresponding author upon request. The authors have annotated the entire data building process and empirical techniques presented in the paper. The data underlying this article are not freely available by agreement with our partners to protect their confidentiality.
